# Biodistribution of Mesenchymal Stem Cell-Derived Extracellular Vesicles in a Radiation Injury Bone Marrow Murine Model

**DOI:** 10.3390/ijms20215468

**Published:** 2019-11-02

**Authors:** Sicheng Wen, Mark Dooner, Elaine Papa, Michael Del Tatto, Mandy Pereira, Theodor Borgovan, Yan Cheng, Laura Goldberg, Olin Liang, Giovanni Camussi, Peter Quesenberry

**Affiliations:** 1 Division of Hematology/Oncology, Brown University, Rhode Island Hospital, Providence, RI 02903, USA; MDooner@lifespan.org (M.D.); EPapa@lifespan.org (E.P.); mdeltatto1@lifespan.org (M.D.T.); mpereira7@lifespan.org (M.P.); Theodor.Borgovan@lifespan.org (T.B.); ycheng@lifespan.org (Y.C.); lrgst6@gmail.com (L.G.); olin_liang@brown.edu (O.L.); 2 Department of Medical Sciences, University of Torino, 10126 Torino, Italy; giovanni.camussi@unito.it

**Keywords:** biodistribution, Extracellular Vesicle, Radiation Injury, Mesenchymal stem cell

## Abstract

We have previously shown that injury induced by irradiation to murine marrow can be partially or completely reversed by exposure to human or murine mesenchymal stem cell (MSC)-derived extracellular vesicles (EVs). Investigation of the biodistribution of EVs in vivo is essential for understanding EV biology. In this study, we evaluated the DiD lipid dye labeled MSC-EV biodistribution in mice under different conditions, including different MSC-EV doses and injection schedules, time post MSC-EV injection, and doses of radiation. DiD-labeled MSC-EVs appeared highest in the liver and spleen; lower in bone marrow of the tibia, femur, and spine; and were undetectable in the heart, kidney and lung, while a predominant EV accumulation was detected in the lung of mice infused with human lung fibroblast cell derived EVs. There was significantly increased MSC-EV accumulation in the spleen and bone marrow (tibia and femur) post radiation appearing with an increase of MSC-EV uptake by CD11b+ and F4/80+ cells, but not by B220 cells, compared to those organs from non-irradiated mice. We further demonstrated that increasing levels of irradiation caused a selective increase in vesicle homing to marrow. This accumulation of MSC-EVs at the site of injured bone marrow could be detected as early as 1 h after MSC- EV injection and was not significantly different between 2 and 24 h post MSC-EV injection. Our study indicates that irradiation damage to hematopoietic tissue in the spleen and marrow targets MSC-EVs to these tissues.

## 1. Introduction

Extracellular vesicles (EVs) have been investigated for many years [1,2,3]. These were initially felt to be cellular debris largely from platelets and erythrocytes [4], but, more recently, intense interest has been focused on their potential for altering target cell fate and tissue repair and regeneration [5]. Vesicles derived from marrow or adipose mesenchymal stem cells (MSCs) have been shown to affect the phenotype and induce healing of many different cell types, including hepatocytes [6], cardiomyocytes [7], pulmonary epithelial cells [8], and renal cells [9], to cite a non-inclusive list. Current knowledge suggests that the repair mechanisms are related to not only their differentiation capacity but also paracrine effects [10,11,12]. The cargo of EVs, including nucleic acids, proteins, and lipids, plays a critical role in EV-mediated stem cell reversal of injured tissues [13,14].

Radiation exposure leads to whole body injury, with variation in the damaging effects based on the dose of radiation received and differential sensitivity of the radiated tissues, especially in the hematopoietic system and the gastrointestinal tract [15,16]. The hematopoietic system is by far the most sensitive tissue to radiation damage, resulting in bone marrow failure or hematopoietic acute radiation syndrome (H-ARS). H-ARS can lead to death within weeks from infection due to loss of neutrophils or hemorrhage due to loss of platelets. We have recently shown that injury induced by irradiation to the murine marrow, either in vitro or in vivo, can be partially or completely reversed by exposure to human or murine MSC-EVs through the inhibition of apoptosis and DNA damage. Moreover, the reversal of radiation damage by MSC-EVs has been shown to be partially mediated by transferring specific EV miRNA subsets [17]. Our studies suggest a unique new approach to radiation mitigation. Therefore, the investigation of MSC-EV biodistribution and recruitment in the irradiated bone marrow injury model is essential for understanding EV biology and their potential therapeutic applications.

Despite the growth of EV research, only a few studies have analyzed EV biodistribution in vivo for EV trafficking [18,19,20]. These studies have shown that damaged, senescent cells internalize labeled EVs more efficiently than non-senescent cells, and that labeled EVs accumulate specifically in the kidneys of the mice with acute kidney injury compared with the healthy controls, suggesting either targeting of the EVs to damaged cells or preferential, more efficient uptake of the EVs by injured cells compared to the healthy cells. No studies to date have reported on MSC-EV’ biodistribution in vivo in a radiation injury model. We evaluated, in vivo, the biodistribution and localization of MSC-EVs in a radiation-exposed murine model. Exosomes and microvesicles are two major populations of extracellular vesicles. Our previous studies have shown that the treatments with the whole population of extracellular vesicles (exosomes and microvesicles) have a better effect on the reversal of radiation damage to bone marrow stem cells both in vitro and in vivo than exosomes or microvesicles alone [17]. Therefore, in this study, we focused on the evaluation of the biodistribution of combined exosome and microvesicle populations in a radiation mouse model under different conditions.

## 2. Results

### 2.1. EV Characterization

**Figure 1:**
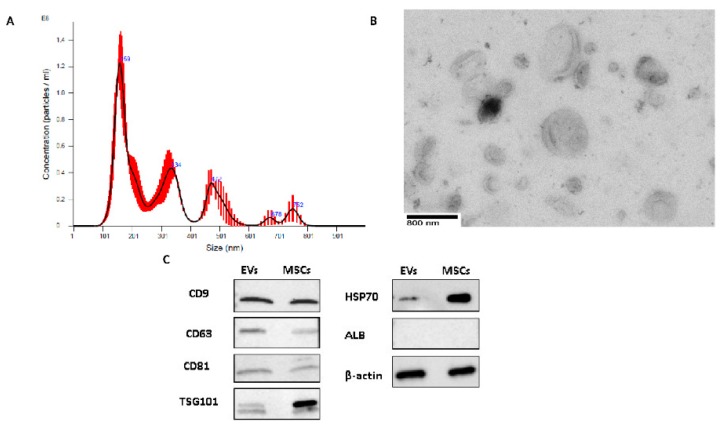
Extracellular vesicle (EV) characterizations. (**A**) A typical profile of human MSC-EV fractions by NanoSight analysis. (**B**) A typical profile of electron micrographs of human MSC-EVs (scale bar = 800 nm; magnification: 14,000×). (**C**) Representative western blot analyses of CD81, CD63, CD9, TSG101, HSP70 ALB, and GAPDH expressions in the lysate of mesenchymal stem cell (MSC)-EVs and MSCs.

Human MSC-EV concentrations and sizes were quantified using NanoSight tracking analysis (NTA) (Figure 1A) and the average size of the EVs was 231.3 ± 124.6 nm. We further verified the size and morphology of EVs by transmission electron microscopy (FEI Morgagni 268) (Figure 1B). Surface epitope protein expression of CD9, CD63, and CD81; internal EV protein expression of GAPDH, HSP70, and TSG101; and a negative control ALB in human MSC-EVs, were evaluated by western blot, and CD81, CD9, CD63, HSP70, TSG101, and GAPDH, but not ALB, were detected in EVs (Figure 1C).

### 2.2. DiD Dye Labelling EVs

The lipophilic near infrared (NIR) dyes, such as DiD or DiR, show weak fluorescence in water, but are highly fluorescent when bound to membranes, and have recently been used for labeling vesicles [18,19,21]. We first evaluated the fluorescence intensity of DiD-labelled EVs directly by fluorescence molecular tomography (FMT) scanning. Serial dilutions of DiD-labelled EVs were loaded on the Parafilm (Fisher Scientific Inc.). A dose-dependent fluorescence intensity of EVs was observed (Supplementary Figure S1A).

**Figure 2:**
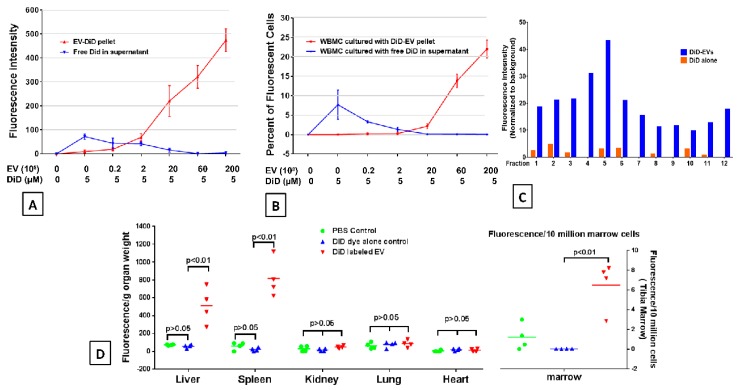
DiD dye labelling EVs. (**A**) Fluorescent intensity of free DiD in supernatants and DiD-EVs pellets after EV labeling and PBS washing were analyzed by FMT. (**B**) Whole bone marrow (WBM) cells were cultured with supernatants and DiD-EV pellets respectively. The percentage of fluorescent WBM cells labeled with free DiD in supernatants and DiD-EV pellets uptake rate by WBM cells were detected by flow cytometry. (**C**) The fluorescence of DiD-labeled EVs and DiD alone—parallel pellets’ gradient fractions. The DiD-labeled EVs and DiD alone: parallel pellets, after PBS washing, were fractionated by density gradient and the twelve fractions were collected and the fluorescent intensity each fraction was analyzed by FMT. (**D**) Fluorescence intensities of hearts, lungs, kidneys, livers, and spleens from mice injected with DiD-labeled EVs were analyzed by FMT. The intensity of fluorescence was expressed as the average of fluorescence intensity (count/energy)/weight of tissue or 10 million bone marrow cells ± SEM. The results were analyzed by one-way ANOVA with a multiple comparisons test. A P value ≤0.05 was considered statistically significant. DiD-labeled EVs versus PBS control or DiD dye control. *n* = 4 mice/group.

In order to exclude the presence of free dye, which could bind to lipids, proteins, or cells, different quantities of EVs were evaluated to ascertain the level at which all dye was bound (Supplementary Figure S1B). A serial titration of EVs from 0, 0.2, 2, 6, 20, 60, and 200 × 10^8^ was incubated in 1 mL PBS with 5 µL 1mM DiD dye at 37 °C for 30 min. The supernatants and DiD-labeled pellets after 100,000 × *g* ultracentrifugation were analyzed for fluorescent signal scanning by FMT-4000 scanner. As we expected, there was an EV-dose-dependent fluorescent signal in DiD-labeled pellets; however, in the supernatants, the fluorescent intensity of the free DiD was very weak (Figure 2A). In order to ascertain free dye labeling to cells, we incubated supernatants with 2 million mouse whole bone marrow cells at 37 °C for 30 min, and to evaluate whether dye labeled EVs (pellets) would be taken up by marrow cells, DiD-labeled EVs pellets were further cultured with 2 million mouse whole bone marrow cells overnight. The fluorescent whole bone marrow cells were analyzed by flow cytometry. For the cells incubated with pellets, the DiD fluorescent cells were not detected until EVs reached 20 × 10^8^. The percentage of DiD fluorescent cells increased as EV dose increased. However, for the cells incubated with supernatants, the highest percentage of DiD-positive cells was detected when cells were incubated with supernatants from the DiD alone preparation, as expected, but decreased when cells were incubated with supernatants from preparations with increasing amounts of EVs, becoming 0% at 20 × 10^8^ (Figure 2B). Our data suggested that to avoid free excess dye existing in DiD-labeled-EVs, the amount of EVs should be at least 20 × 10^8^ when labeled with 5 µL of 1mM DiD dye. We further evaluated the DiD labelled EVs and DiD alone in parallel pellets by sucrose-density gradient fractionation. The fluorescence was detected in all twelve fractions with a peak at factions 4 and 5 (Figure 2C). This coincides with our previous work, having shown that EVs widely distributed among all twelve fractions, being heterogeneous in their quantity and composition [22]. A very weak fluorescence signal was also detected in fractions from the DiD dye alone pellet gradient. Thus, the EV sucrose-density gradient fractionation analysis suggested that there is detectable free dye or dye aggregates in the DiD-labeled EVs. We evaluated that next in in vivo studies. 2 × 10^10^ EVs were labeled with 5 µL of 1 mM DiD, dye as described above. EV-free PBS was incubated with the same amount of DiD dye as a dye alone control and processed in parallel with the EV-labeled samples. After PBS washing and ultracentrifugation, the supernatant was removed and the pellets on the bottom of tubes from the DiD dye labelled EVs and DiD dye alone control were resuspended with 1 mL of PBS respectively. Then, 2 × 10^9^ DiD-labelled EVs, the DiD alone control (same volume of DiD dye), and the PBS vehicle control were each injected into 500 cGy radiated C57BL/6 mice by tail vein injection. Six hours post injection, hearts, lungs, kidneys, livers, spleens, and bone marrow from mice were dissected then imaged using the FMT 4000™ fluorescence tomography imaging system for DiD-labeled EV quantification. There were no fluorescent signals in any organs from mice injected with DiD alone or the PBS control, but there were very strong fluorescent signals in livers, spleens, and bone marrow from mice injected with DiD-labeled EVs compared to DiD alone or PBS vehicle control mice (Figure 2D and Supplementary Figure S2). The MSC-EV internalization in spleen cells was evaluated by confocal microscopy (Supplementary Figure S3). Thus, although DiD-labeled EVs prepared by current methods could not absolutely exclude the presence of free dye or free dye aggregates, our in vitro and in vivo data (DiD dye alone parallel control) indicate that the free dye or free dye aggregates are undetectable or ignorable in cells or tissues.

### 2.3. EVs Accumulate in Radiated Livers, Spleens, and Bone Marrow

We then evaluated the biodistribution of DiD-labeled MSC-EVs in a radiated mouse model, as previously described [17]. Mice were exposed to 500 cGy radiation, and 24 h later, 2 × 10^9^ of DiD-labeled human MSC-EVs were injected into mice by tail vein injection. Then, using the FMT 4000™ fluorescence tomography, we quantified the fluorescence signal in the heart, lung, spleen, kidney, liver, tibia, femur, and spine 6 h after MSC-EV injection. We found the DiD-labeled MSC-EVs appeared most in the liver and spleen, less in bone marrow, and were undetectable in the heart, kidney, and lung. We confirmed our previous work [17], as there was a significant increase in EV accumulation in spleens and the bone marrow from tibias and femurs post radiation compared to those organs from non-irradiated mice.

**Figure 3:**
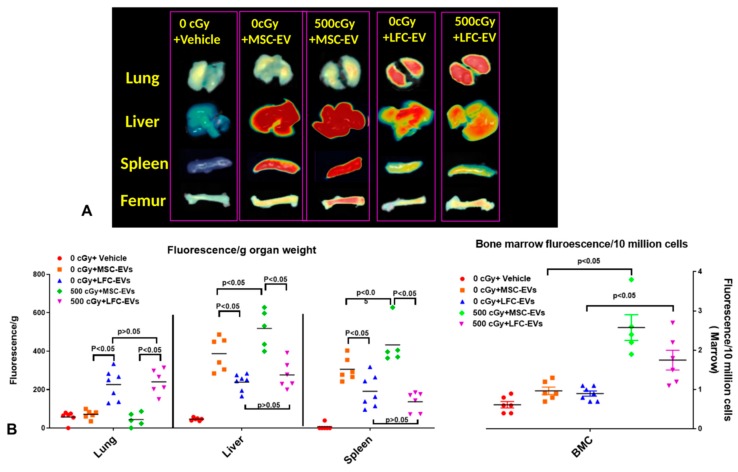
MSC-EV and lung fibroblast cells derived EV (LFC-EV biodistribution in mice. (**A**) Representative tissue organ FMT images from mice infused with DiD-labeled MSC-EVs and FLC-EVs. (**B**) Quantification analysis of fluorescence intensity in tissues (*n* = 5 or 6 mice/group). The results were analyzed by one-way ANOVA with a multiple comparisons test.

To further determine whether there is a specific accumulation of human MSC-EVs in livers, spleens, and bone marrow from radiation-exposed mice, we next evaluated the biodistribution of DiD-labeled human lung fibroblast cell (Lonza, Walkersville, MD number: CC-2512) derived EVs (LFC-EVs) in the same radiation mouse model as described above. Again, there was undetectable LFC-EV accumulation in both the heart and kidney after EV injection, as we found with MSC-EV injection. However, we found that there was a significant LFC-EV accumulation in lung tissue, whereas that was undetectable with MSC-EVs for mice with or without radiation, and the amount of LFC-EV accumulation was much lower compared to MSC-EVs in the liver and spleen from non-irradiated mice (Figure 3A,B). Moreover, there was no significant alteration of LFC-EV accumulation in the liver, spleen, or lung after radiation exposure, where the MSC-EV accumulations in the spleen and liver were significantly increased. Interestingly, we found there was also a significant increase of LFC-EV accumulation in bone marrow after radiation (Figure 3A,B). The above data suggested that EVs from different cell sources could influence the EV-biodistribution, as reported by others [18], and confirmed our previous study and suggests a specific accumulation of MSC-EVs at the site of the liver, spleen, and bone marrow after radiation exposure.

### 2.4. MSC-EVs Are Internalized in Different Immune Cells

**Figure 4:**
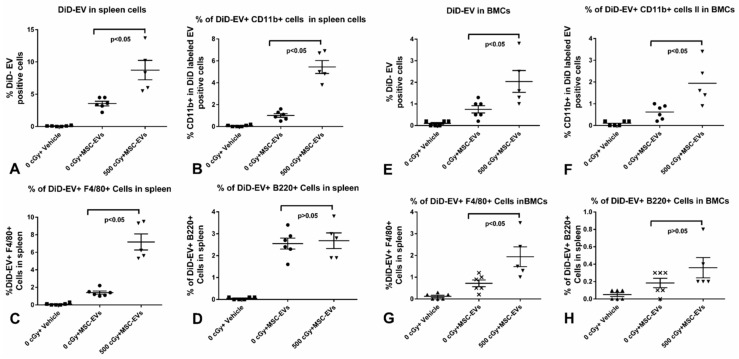
MSC-EV uptake in different immune cells determined by flow cytometry. The alteration of MSC-EV uptake in spleen (**A**) and bone marrow (**E**) from mice post radiation exposure. The alteration of MSC-EV uptake by CD11b+ cells in the spleen (**B**) and marrow (**F**), F4/80+ cells in the spleen (**C**) and bone marrow (**G**), and B220+ cells in spleen (**D**) and marrow (**H**) from mice post radiation exposure. *n* = 5 or 6/group. The one-way ANOVA with multiple comparisons test was used as statistical analysis.

We further analyzed the DiD-labeled MSC-EV uptake in the spleen and bone marrow by flow cytometry assay. DiD-labeled human MSC-EVs were injected into 24 h-post 500 cGy irradiated mice, and at 6 h post EV injection, the mice were sacrificed, and a single cell suspension was prepared from each mouse spleen and femur bone marrow. We found that there was a significant increase MSC-EV uptake in the spleen compared to bone marrow, with the uptake rates 3.57% ± 0.35% and 0.75% ± 0.17%, respectively, in non-radiated mice. The spleen and bone marrow cells presented an increased MSC-EV uptake after radiation from 3.57% ± 0.35% to 8.74% ± 1.50% and 0.75% ± 0.17% to 2.04% ± 0.51%, respectively (Figure 4A,E). These data confirm that there is a specific accumulation of MSC-EVs at the site of spleen and bone marrow.

To determine the cell populations that were taking up DiD-labeled MSC-EVs, CD11b+, B220+, and F4/80+ cells in the spleen and bone marrow were further evaluated by flow cytometry assay. CD11b+, B220+, and F4/80+ cells were presented as the population of myeloid, lymphoid, and macrophage predominant cell populations, in the spleen and bone marrow. MSC-EV uptake (DiD +) in the spleen CD11b +, F4/80 +, and B220+ cells from non-irradiated mice was 1.017% ± 0.16%, 1.4% ± 0.18%, and 2.55% ± 0.25%, respectively (Figure 4B–D). After radiation, the CD11b+, and F4/80+ cells from the spleen showed an increased MSC-EV uptake at 5.44% ± 0.59%, and 7.18% ± 0.92% compared to non-irradiated mice at 1.017% ± 0.16% and 1.4% ± 0.18%, respectively (Figure 4B,C). However, the B220+ cell uptake of MSC-EVs in the spleens from mice exposed to radiation did not show alteration with 2.68% ± 0.36% compared to 2.55% ± 0.25% in non-irradiated mice (Figure 4D). And again, the alteration of update of MSC-EV in CD11b+, B220+, and F4/80+ cells in bone marrow from mice exposed to radiation showed a similar pattern as in the spleen (Figure 4F, G and H). Thus, our data suggested that the specific MSC-EV accumulation in spleen and bone marrow after radiation might be due to increased uptake of MSC-EVs by CD11b+ and F4/80+ cells in spleen and bone marrow.

### 2.5. Accumulation of EVs in Bone Marrow is Dependent on the Dose of Radiation Exposure

**Figure 5:**
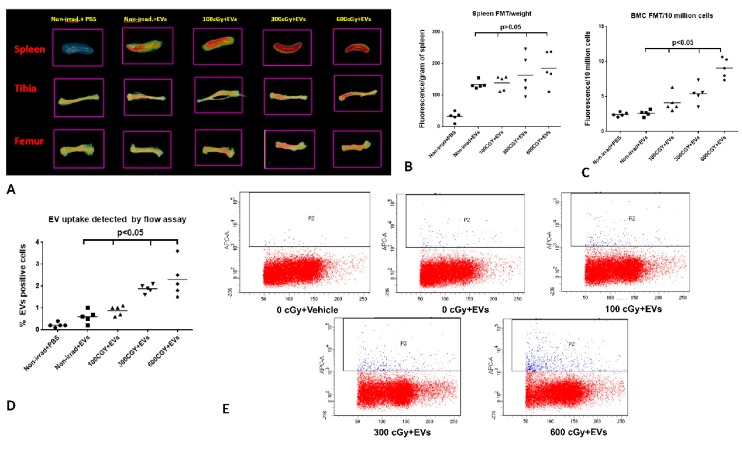
EV biodistribution in spleens, tibias, and femurs. (**A**) Representative images of a spleen and tibia and femur bone marrow from mice with 0 or 100, 300, and 600cGy radiation exposure at 6 h after DiD-EVs injection. *n* = 5 mice/group. (**B**) DiD-labeled-EV accumulation in the spleen. (**C**) Quantification of DiD-labeled EVs within bone marrow. (**B,C**) The intensity of fluorescence was expressed as the average of fluorescence intensity (count/energy)/weight of tissue or 10 million bone marrow cells ± SEM. The fluorescence intensity in bone marrow from EV treated animals with or without radiation were analyzed by ANOVA. A P value ≤0.05 was considered statistically significant (**D**) Uptake of human MSC-EVs by Murine bone marrow cells. After FMT analysis, the bone marrow cells were flushed from one tibia and one femur, and DiD positive bone marrow cells were detected by flow cytometry. The percentage of DiD-positive BMCs from EV treated animals with or without radiation was analyzed by ANOVA. A P value ≤0.05 was considered statistically significant (**E**) Representative flow cytometric analysis of MSC-EV uptake by Murine bone marrow cells.

Next, we investigated whether the dose of radiation exposure affects the EV biodistribution in radiated mice. For these experiments, mice were exposed to 100, 300, or 600 cGy (*n* = 5 mice/group), and that was followed by injection with 2 × 10^9^ of DiD-labeled human MSC-EVs by tail vein 24 h after radiation. DiD dye EV labeling was performed as described above. At 6 h post EV injection, the mice were sacrificed, and hematopoietic organs were harvested and the fluorescence signal within tissues was analyzed using FMT 4000 system. Again, we confirmed that there was significantly increased EV accumulation in the spleen and bone marrow from tibias and femurs post radiation, especially those exposed to 600 cGy compared to those organs from non-irradiated mice (Figure 5A–C). Moreover, bone marrow tissue fluorescence progressively increased with increasing radiation dose after injection with DiD-labelled human MSC-EVs, and there was an increasing trend in the spleen. Our result further confirmed that these MSC-EVs specifically accumulated at the site of spleen and marrow post radiation exposure and the bone marrow accumulation was dependent on the dose of radiation exposure.

We also evaluated EV uptake by bone marrow cells from tibia and femur in the above experiments. The bone marrow cells from the tibia and femur were flushed out 6 h post EV injection, and the EV uptake rate of bone marrow cells (DiD positive cells) was analyzed by flow cytometry. The EV uptake in bone marrow from tibias and femurs with 100, 300, and 600 cGy radiation exposure was 0.88% ± 0.10%, 1.88% ± 0.09%, and 2.3% ± 0.36%, respectively. This was significantly increased when compared to those organs from non-irradiation-exposed mice at 0.60% ± 0.13% after DiD-labeled EV injection (*p* < 0.05, Figure 5D,E). This suggested the accumulation of EVs at the site of injury might be due to increased EV uptake by radiation-injured bone marrow cells. 

### 2.6. Accumulation of EVs in the Spleen and Bone Marrow Is Dependent on the Dose of EVs Administered

**Figure 6:**
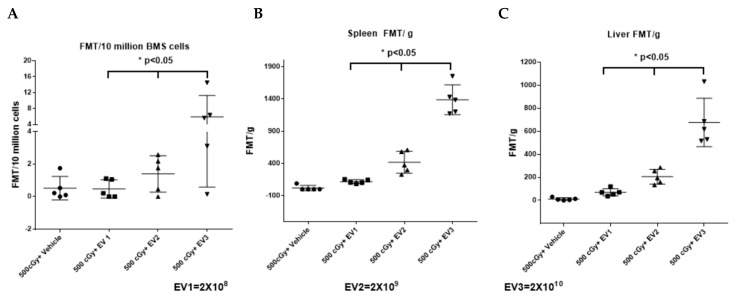
A dose-dependent increase in tissue uptake in mice by systemically administrated EVs in the (**A**) bone marrow, (**B**) spleen, and (**C**) liver. The intensity of fluorescence was expressed as the average of fluorescence intensity (count/energy)/weight of tissue or 10 million bone marrow cells ± SEM, *n* = 5/group. One-way ANOVA with a multi-comparison test was performed to evaluate the fluorescence intensities in bone marrow, spleen, and liver tissues from animals that received three different doses of EV respectively. * *p* < 0.05.

To assess the MSC-EV biodistribution, three different doses of DiD-labeled MSC-EVs at 2 × 10^8^, 2 × 10^9^ and 2 × 10^10^ were injected into mice by tail vein at 24 h post 500 cGy-whole body radiation exposure. The livers, spleens, femurs, and tibias were collected and the DiD-labeled EVs within tissues were analyzed using the FMT 4000 system. There was a dose-dependent increase in EV accumulations in the liver, spleen, and bone marrow 6 h after mice received intravenously-administered EVs (Figure 6).

### 2.7. The Time Course of EV Distribution in Mice after EV Injection 

**Figure 7:**
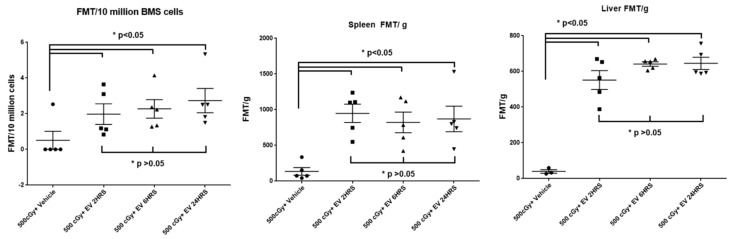
The fluorescence signals in the liver, spleen, and bone marrow from 500 cGy radiation exposed mice at 2, 6, and 24 h post-systemically administrated EVs. The intensity of fluorescence is expressed as the average of fluorescence intensity (count/energy)/weight of tissue or 10 million bone marrow cells ± SEM, *n* = 5/group. The results were analyzed by one-way ANOVA with a multi-comparison test, * *p* < 0.05 EV treated groups compared to vehicle control group.

**Figure 8:**
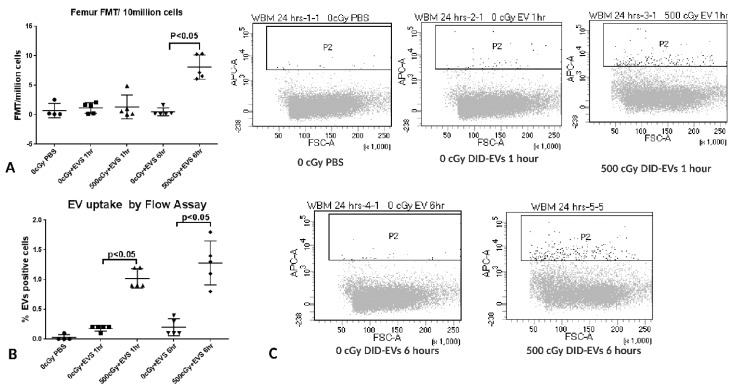
EV accumulation in tissues from 0 or 500 cGy radiation exposed mice at 1 and 6 h post-systemically administered EVs. (**A**) EVs in the bone marrow cells determined by FMT. The intensity of fluorescence was expressed as the average of fluorescence intensity (count/energy)/10 million bone marrow cells ± SEM. (**B**) The DiD positive bone marrow cells harvested from femurs were detected by flow cytometry. The one-way ANOVA with a multiple comparisons test was performed. A P value ≤0.05 was considered statistically significant, 500 cGy + EVs 1 h, or 500 cGy + EVs 6 h versus non-irradiated + PBS. (**C**) Flow cytometry diagrams of DiD-positive bone marrow cells from 0 or 500 cGy radiation exposed mice at 1 and 6 h post EV injection. *n* = 5 mice/group.

To investigate the time course of the EV distribution post EV injection, mice were intravenously injected with 2 × 10^9^ of DiD-labeled human MSC-EVs by tail vein 24 h post-500 cGy irradiation. At 2, 6, and 24 h post EV injection, spleens, livers, tibias and femurs were harvested from sacrificed mice, and the fluorescent signals within tissues were analyzed using the FMT 4000 system. The fluorescent signals in the spleen, liver, tibia and femur were detected 2 h post EV injection and persisted for 24 h without significant change (Figure 7). We also evaluated EV distribution at 1 and 6 h post EV injection by FMT and flow cytometry assays. Similarly, the fluorescent signals in the liver and spleen were able to be detected at 1 and 6 h. Although the fluorescent signal in bone marrow was not detected by FMT 1 h post EV injection, it was detected by flow cytometry at this early time point (Figure 8).

### 2.8. EV Biodistribution in Mice after Single or Triple Doses of EV Injections

**Figure 9:**
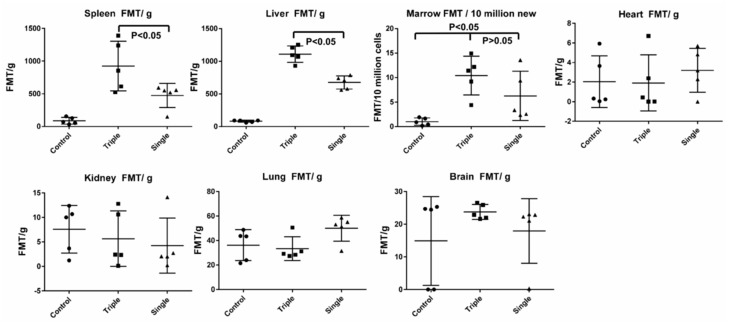
EV accumulation in tissues from mice with single versus triple EV injections. The intensity of fluorescence was expressed as the average of fluorescence intensity (count/energy)/weight of tissue or 10 million bone marrow cells ± SEM; *n* = 5 mice/group. The results were analyzed by one-way ANOVA with multiple comparisons test; A P value ≤0.05 was considered statistically significant, triple versus single dose in spleen and liver.

We further evaluated the EV biodistribution in mice after single versus triple EV injections. Mice were irradiated with 500 cGy-whole body irradiation, and then 24 h later, were injected with either 2 × 10^9^ EVs—one dose, or 2 × 10^9^ EVs daily—three doses. The brain, heart, lung, liver, spleen, intestine, kidney, and femur were dissected and analyzed for fluorescent signal scanning. There were significantly increased EV accumulations in the liver and spleen and there was an increasing trend in bone marrow with triple EV injections, compared to a single injection. There was no detected fluorescent signal in the brain, heart, kidney, lung, or intestines with either triple or single EV injection (Figure 9).

## 3. Discussion

In our previous published studies, we have shown that human or murine MSC-EVs rescue radiation damage to bone marrow cells in a murine model [17]. We found that the MSC-EV treatment could significantly improve the bone marrow stem cell engraftment capacity from radiation-exposed mice up to 9 months post-irradiation. It also increased FDC-P1 cells (a murine factor-dependent myeloid progenitor cell line) proliferation and inhibited apoptosis and DNA damage. We also found that there was an increase in MSC-EV accumulation in bone marrow and spleen tissues after radiation, which might facilitate MSC-EV radiation reversal capacity. Our previous work also showed that MSC-derived vesicles, which include microvesicles and exosomes (isolated by differential centrifugation at 2000 × *g* for 30 min, followed at 100,000 × *g* for 1 h), have a better healing effect on the reversal of radiation damage both in vitro and in vivo than microvesicles (isolated by differential centrifugation at 2000 × *g* for 30 min, followed at 10,000 × *g* for 1 h), or exosomes (isolated by differential centrifugation at 2000 × *g* for 30 min, followed at 10,000 × *g* for 1 h; then, remove supernatant and collect vesicle with another centrifugation at 100,000 × *g* for 1 h) alone. Thus, in this study, we focused on the investigation of the biodistribution of combined microvesicle and exosome populations in mice under different conditions, including EV doses and injection schedules, time post EV injection, and different doses of radiation.

In this study, we have shown that labeled MSC-EVs accumulated in the liver, spleen, and bone marrow of radiation-exposed mice, but no detectable signal was found in heart, kidney, or lung, while no signal was detected in any organs in DiD alone or PBS control mice. Interestingly, we found that the biodistribution of LFC-EVs is different from MSC-EVs, with that of the LFC-EVs showing a predominant accumulation in the lung and less accumulation in spleen and liver; moreover, there was no significant LFC-EVs accumulation change in the spleen or liver after radiation. This suggested a specific accumulation of MSC-EVs at the site of liver, spleen, and bone marrow radiation injury and that the EV biodistribution might be determined by cell source and targeting tissue. We found EV accumulation in bone marrow, the liver, and the spleen could be detected as early as 1–2 h-post EV injection and was maintained up to 24 h post-injection. Our data also showed that both increasing the number of EV injections and dose of EVs per injection, and increasing doses of irradiation, resulted in increased EV uptake in the liver, spleen, and bone marrow. Moreover, the radiation dose-dependence suggested a specific accumulation of EVs at the site of injury. The EV uptake in marrow cells increased progressively from 100 to 600 cGy, indicating that radiation injury may target the EVs to the marrow cells. The mechanisms underlying the organ specificity of EV biodistribution are largely unknown and could be affected by a multitude of factors, including cell source, injection route, and EV surface markers [20]. We also found that there is an increase of uptake of MSC-EV by CD11b+ and F4/80+ cells but no change in B220 + cells in the spleen and bone marrow after radiation, suggesting the MSC-EV accumulation in spleen and bone marrow relate to the increased uptake by monocytes, neutrophils, or macrophages. A recent study has shown that the exosome uptake in monocyte/macrophages is mediated by the scavenger receptor. By using dextran sulfate, a competitive inhibitor for scavenger receptor, the biodistribution of EVs in mice was significantly changed [23]. It is known that MSCs localize to the kidney by utilizing CD44, following acute tubular injury; therefore, the accumulation of EVs at the site of injury might be due to EVs carrying the same membrane receptors as MSCs [19]. It will be interesting to investigate the effect of blockade of SR-A with dextran sulfate on EV biodistribution in the radiation bone marrow injury murine model. Additionally, this work demonstrates the in vivo distribution of extracellular vesicles combined with exosome and microvesicle populations when injected intravenously. Other work has shown that different routes of administration can alter the distribution to the liver, spleen, and pancreas [18]. Therefore, it will be of interest to determine how altering the route of EV administration or administration exosomes or microvesicles alone, following irradiation, may alter EV homing in this model.

Labeling EVs with lipophilic dyes, such as PKH, DiD, and DiR has been widely used recently for tracking the EV biodistribution in animals [18,19,23,24,25]. In this study, we directly labeled EVs with DiD dye, and that was followed by ultracentrifugation purification through 30 mL PBS washing twice. We have successfully demonstrated the MSC-EV biodistribution in radiation exposure mouse model. Although we could not exclude the presence of free dye or free dye aggregates in DiD dye labeled EVs, by analyzing the fluorescence both in pellets and supernatants from a series of increasing amounts of EVs with DiD or DiD alone parallel control, and bone marrow cells exposed to pellets and supernatant, we found that the free dye or free dye aggregates existing in DiD-labeled-EVs was inadequate to give the observed tissues or cellular fluorescence. Our data suggested that to avoid free excess dye existing in DiD-labeled-EVs, the amount of EVs should be at least 20 × 10^8^ when labeled with 5 µL 1mM DiD dye. By further analysis of fluorescence of the floating fractions from DiD labelled EVs and DiD alone parallel pellets, after sucrose-density gradient ultracentrifugation, we found that the fluorescence was detected in all twelve fractions with a peak at factions 4–6, which were enriched with exosomes. This coincides with our previous work having shown that mixtures of exosome and microvesicle populations of EVs widely distributed among all twelve fractions were heterogeneous in their quantity and composition [22]. These experiments indicated that there is only very little amount of free dye or free dye aggregates in our labeled EVs, and that the observed fluorescence in tissues is due to labeled EVs, which was further confirmed by the following experimental results: First, no fluorescent signal was detected in any organs in DiD alone parallel control mice after DiD-labeled EV injection. Second, we observed the different biodistribution profile between MSC-EVs and LFC-EVs (predominantly accumulated in the lung) in healthy mice. Moreover, there was no significant alteration of LFC-EV accumulation in the liver, spleen, or lung after radiation exposure, whereas the MSC-EV accumulations in the spleen and liver were significantly increased, indicating that EV homing to tissues is influenced by the cell sources of EVs. We also found that the accumulation of MSC-EVs in bone marrow is related to the dose of radiation exposure and the specific MSC-EV accumulation in the spleen and bone marrow after radiation are related to the increased uptake of MSC-EV by CD11b+ and F4/80+ cells in spleen and bone marrow but not B220 +cells. More importantly, in our previous published study, we have shown the MSC-EVs could reverse radiation bone marrow damage both in vitro and in vivo by inhibition of apoptosis and DNA damage [17]. Thus, the above data suggest the MSC-EV biodistribution in the radiation mouse model is a specific biological activity and it did not relate to free dye or free dye aggregates nonspecific binding to cells or tissue. Our future works will focus on the investigation of the mechanism of MSC-EV biodistribution in this model.

In conclusion, the specific localization of human MSC-EVs to injured hematopoietic tissue, but not the normal hematopoietic system, may facilitate MSC-EV-mediated reversal of radiation damaged bone marrow cells. Understanding the biodistribution of EVs in healthy and injured tissues will be useful for optimizing their potential in therapeutic applications. 

## 4. Material Methods

### 4.1. Experimental Animals

We used 6–8 weeks old male C57BL/6 mice in this study (Jackson Laboratory, Bar Harbor, ME, USA) and followed the protocols approved by the Institutional Animal Care and Use Committee at Rhode Island Hospital (Lifespan Animal Care and Use Protocol#0131-16, 7 July 2016). All mice were housed in micro-insulator cages in the animal facility for at least 1 week for acclimation prior to experimental use. We used CO_2_ inhalation followed by cervical dislocation to euthanize the animals.

### 4.2. Cell Culture and EV Isolation

Human mesenchymal stem cells (MSC) were purchased from Lonza (Walkersville, MD, USA, #PT-2501) and cultured in a 175 cm^2^ flask with 25 mL mesenchymal stem cell media (Lonza, Walkersville, MD, USA, #PT-3001) in a humidified incubator at 37 °C with 5% CO_2_, according to manufacturer’s protocol. The cell propagation was limited to passage 10.

After reaching 80–90% confluency (about 1.5–2 million cells/flask), human MSCs were washed with 15mL PBS followed by adding 25 mL serum-free RPMI (Life Technologies) medium for another 24 h. MSC-derived EVs were collected from the culture medium by differential centrifugation at 2000 × *g* for 30 min to remove cell debris, and then, 100,000 × *g* for 1 h, collecting of the 100,000 × *g* pellet after. EVs were stored in PBS supplemented with 1% DMSO at −80 °C until EV DiD dye labeling. We used WX Ultra Centrifuge with Sorvall AH-629 rotor.

### 4.3. EV Characterization

The size distribution and number of EVs were determined by nanoparticle tracking analysis (NTA) using NanoSight NS500 (Malvern Instruments, Malvern, UK) equipped with a Syringe Pump and NTA software. The analysis settings were first tested against known control beads (Malvern Instruments) and the acquisition optimized for each sample. Sample dilutions were made to particle concentrations in the range of 1 × 10^8^ particles per mL, in accordance with the manufacturer’s recommendations. All samples were analyzed in triplicate and further evaluated by electron microscopy as previously described [17]. Briefly, EVs were fixed in 50 µL of 2% paraformaldehyde and transferred onto Formwar-carbon-coated electron microscopy grids. After membranes were covered for 20 min and washed with PBS, EVs were then fixed in 1% glutaraldehyde and contrasted in 4% uranyl acetate. The samples on grids were observed under the transmission electron microscope (FEI Morgagni 268) at 80 kV. The markers on EVs including CD81(EXOAB-CD81A-1), CD9(EXOAB-CD9A-1), TSG101(MA1-23296), GAPDH(AM4300), CD63(sc-5275), heat shock protein 70(HSP70) (sc-59560), and Albumin(ALB)(sc-58688) from System Biosciences (Mountain View, CA, USA), Thermofisher (Waltham, MA USA), and Santa Cruz Biotechnology Inc (Dallas, TX, USA) were evaluated by western blot. 

### 4.4. EV Labeling with DiD Lipid Dye

EVs were incubated with 1 mL PBS with 5 μM Vybrant™ DiD Cell-Labeling Solution (ThermoFisher Scientific, Waltham, MA USA) for 30 min at 37 °C. We washed labeled EVs twice with 30 mL 1xPBS by ultracentrifugation at 100,000 × *g* for 1 h to remove excess dye (Sorvall AH-629 rotor, WX Ultra Centrifuge) and resuspended them in 1 × PBS for EV tail vein injection.

### 4.5. Fluorescence Molecular Tomography (FMT)

The EV biodistribution in organs of animals was investigated by FMT as previously described [17]. Organs were dissected from mice after EV injection and were then imaged using the FMT 4000™ imaging system (PerkinElmer, Waltham, MA, USA) to collect fluorescence reflectance images. The quantification of a three-dimensional fluorescence signal within tissues was analyzed using FMT 4000 system software (TrueQuant v3.0, PerkinElmer, Waltham, MA, USA) by reconstruction of the collected fluorescence data. Three dimensional regions of interest (ROI) in tissues were selected to measure the fluorescence intensity of EVs in dissected tissues. Unless otherwise stated, for the fluorescence intensity of organs, we normalized the fluorescence intensity by organ weight (per gram tissue). For fluorescence intensity of bone marrow, the bone marrow cells flushed from tibias and femurs were counted by hemocytometer after detection of fluorescence by FMT and we normalized the fluorescence intensity to 10 million bone marrow cells. The average of fluorescence intensity (count/energy) ± the standard error of the mean (SEM) is presented as fluorescence intensity of tissues.

### 4.6. Sucrose Density Gradient Ultracentrifugation

Sucrose gradient was prepared as previously described [22,26]. Solutions of 10%, 16%, 22%, 28%, 34%, 40%, 46%, 52%, 58%, 64%, 70%, and 90% sucrose (Sigma-Aldrich) were prepared with 1 × PBS. We resuspended the 100 µL DiD-labeled EVs with 1 mL of 90% sucrose solution and transferred it to a 13 mL ultracentrifuge tube (Beckman Coulter, Brea, CA, USA). Sucrose gradient were overlain on the top of EVs, starting with from 1 mL of 70% to 10% sucrose solutions. Ultracentrifugation was carried out at 4 °C at 100,000 × *g* for 16 h (Sorvall AH-629 rotor, WX Ultra Centrifuge). Twelve 1 mL gradient fractions were collected starting from the top of the gradient for the fluorescence detection by FMT scanning. Fraction 4 and 5 were enriched for exosomes.

### 4.7. Radiation Injury Murine Model

In this study, the times of EV injection in all experiments were scheduled for 24 h post whole-body radiation exposure (07:00) (Gammacell 40 Exactor, Cesium 137 source irradiator, 0.94–0.96 Gy/min). We evaluated the effects of EV bio-distribution in mice exposed to different doses of whole-body irradiation under varied conditions, including different doses of EVs infused, different times post EV injection, and single EV injection vs. triple dose EV injection. The organs (lung, spleen, kidney, liver, heart, femur, and tibia bone marrow) were immediately dissected after mice were sacrificed for EV bio distribution analysis by fluorescence molecular tomography. In addition, the uptake of EVs by bone marrow cells was assessed by flow cytometry (BD LSR II flow cytometer with BD FACSDiva™ software, BD Biosciences).

### 4.8. Spleen Single Cell Suspension Preparing, Staining, and Imaging

Six hours post DiD-labeled-EV injection, spleens from mice were dissected and transferred into a 35 mm culture dish with 2 mL cold PBS for single cell suspension preparations. Briefly, the spleen was gently crushed with the flat-back end of piston/plunger from a sterile 3 cc syringe, and then, the supernatant and tissue from the dish were transferred to the primed 40 μm cell strainer, the strainer washed with 10 mL of PBS three times, and cells collected by centrifugation at 300 × *g* for 10 min. A cell suspension was prepared at 0.5 × 10^6^ cells/mL with 1% FBS of PBS and the cells were mounted on the microscope slide by loading 200 µL cells suspension into a Cytospin cuvette and that was spun at 300 rpm for 10 min. The cells were fixed with 10% formaldehyde for five minutes and rinsed three times in PBS for 5 min each; then, the slides were incubated with Alexa Fluor^®^ 488 Phalloidin (Cell Signaling Technology, Danvers, MA, USA) at a 1:20 dilution in PBS for 15 min at room temperature rinsed once with PBS, and placed on coverslip slides with ProLong^®^ Gold Antifade Reagent (ThermoFisher Scientific, Waltham, MA USA). The EV internalization in spleen cells was then identified by confocal fluorescence Nikon C1si confocal microscope (Nikon Inc., Mellville, NY, USA).

### 4.9. Flow-Cytometry Analysis

To detect the EV uptake by different cell populations in the spleen and bone marrow, the bone marrow cells from the tibia and femur were flushed into PBS with 1% FBS, and the spleen single cell suspensions were prepared as described above. The cells were then incubated with anti-CD11b PE (eBioscience for detection of Monocytes, macrophages, natural killer cells, dendritic cells, neutrophils, some memory B cells, and NK cells), anti-B220 Pacific Blue (ThermoFisher, for detection of B cell lymphocytes), and anti F4/80+ APC (eBioscience for detection of macrophages and monocytes) for 30 min at room temperature, which was followed by lysing RBC with BD Pharm Lyse™ lysing solution. The DiD-labeled EV+ with CD11b+, B220+, and *F4/80*+ cells were detected by the BD LSR II flow cytometer (BD Biosciences).

### 4.10. Statistics

All study data are expressed as means ± SEMs. The data were statistically analyzed by *t-*tests or ANOVAs with multi-comparison tests using Prism 6.0 (GraphPad Software, Inc. San Diego, CA USA). A *p*-value of < 0.05 was considered statistically significant.

## Supplementary Materials

Supplementary Materials are available online at https://www.mdpi.com/1422-0067/20/21/5468/s1.

## Abbreviations

MSC	Mesenchymal stem cell
EVs	Extracellular vesicles
H-ARS	Hematopoietic acute radiation syndrome
LFC	Lung fibroblast cell
FMT	Fluorescence molecular tomography
