# Supplementary material for: Biodistribution of Mesenchymal Stem Cell-Derived Extracellular Vesicles in a Radiation Injury Bone Marrow Murine Model
Source: Int J Mol Sci. 2019 Nov 2;20(21):5468. doi: 10.3390/ijms20215468 (PMC6861905; doi:10.3390/ijms20215468)
Supplement: Supplementary file 1 [file ijms-20-05468-s001.zip › ijms--supplementary/supplemental Figure Legends.docx]

**Supplemental Figure 1 Flowchart of EV labeling**

**(A)**Fluorescence images of extracellular vesicle (EV) dilutions **(B)** Flowchart outlining the steps of EV DiD labeling and evaluation

**Supplemental Figure 2 FMT images**

Representative fluorescence molecular tomography (FMT) images from liver, spleen, heart, lung, kidney and bone marrow in tibia of mice injected with DiD labeled EVs, EV-free DiD alone parallel control, PBS-Vehicle control.

**Supplemental Figure 3 The confocal imaging of MSC-EVs internalization in spleen cells**

The mouse spleen tissues were harvested 6 hours after tail vein injection with DiD labeled MSC-EVs, and the spleen cytological slides from single spleen cell suspension prepared by cytospin were evaluated for EVs internalization in spleen cells using confocal fluorescence confocal microscope**. A)** Representative confocal images of fluorescently staining the cytoskeleton F-actin labeled with Alexa Fluor 488 phalloidin (green). **B)** Representative confocal images of DiD labeled MSC-EVs in spleen cell. **C)**  Differential interference contrast (DIC) image. D) The merge image highlights localization (red) of EV. **E)** Three-dimensional presence of DiD labeled EV on the surface and inside the spleen cell. Z series sections were collected at 0.15µm with a 100x Plan Apo lens and Z-stack image created by merging serial scans of thick tissue section (20 µm). The images were viewed under 300x magnification confocal laser scanning microscopy.
